# A comparative assessment of color stability among various commercial resin composites

**DOI:** 10.1186/s12903-023-03515-9

**Published:** 2023-10-24

**Authors:** MB Uctasli, Sufyan Garoushi, M Uctasli, PK Vallittu, L Lassila

**Affiliations:** 1 Department of Restorative Dentistry, Faculty of Dentistry, University of Gazi, Ankara, Turkey; 2 Department of Biomaterials Science and Turku Clinical Biomaterial Center - TCBC Institute of Dentistry, University of Turku, Turku, Finland; 3 Department of Restorative Dentistry and Cariology, Adhesive Dentistry Research Group Institute of Dentistry, University of Turku, Turku, Finland; 4 Wellbeing Services County of South-West Finland, Turku, Finland

**Keywords:** Color stability, Repolishing, Resin composite, Coffee, EverX Flow, SDR flow+

## Abstract

**Objectives:**

The aim was to evaluate the color stability of six commercial restorative resin composites after being exposed to commonly consumed beverages. Repolishing impact on the stained composite was also assessed.

**Methods:**

One-hundred and fifty disc specimens (8 mm diameter & 3 mm thickness) were prepared from Filtek™ Universal Restorative, SDR flow+, everX Flow, G-ænial A’CHORD, G-ænial Universal Flo and G-ænial Universal Injectable. To assess the color stability in five various beverages, 25 specimens from each material were randomly distributed into five groups (n = 5), according to the utilized staining solution. Group 1: distilled water, Group 2: coffee, Group 3: red wine, Group 4: energy drink, Group 5: coke. The color changes (∆E) for all materials were measured using spectrophotometer at the baseline, after 84 days of staining and after repolishing. Data was collected and analyzed using ANOVA (α = 0.05).

**Results:**

Both material type and staining solution had a significant effect on the color stability of specimens (p < 0.05). Compared to other beverages, the color value of the specimens submerged in coffee and wine showed the most statistically significant (p < 0.05) mean ∆E. SDR flow + in coffee and wine presented the highest ∆E when compared to other tested materials (p < 0.05). After staining of the composites, repolishing was successful in lowering the ∆E value.

**Conclusions:**

All the beverages had an impact on the color stability of the tested resin composites, with coffee and wine demonstrating the most significant effects. The variations in color stability varied depending on the specific material utilized. Dentists should possess awareness regarding the chemical interactions that occur between different beverages and various types of resin composites. Additionally, repolishing serves as an effective technique for eliminating surface discoloration in composite restorations.

## Introduction

Advancements in minimally invasive dentistry have led to the emergence of resin-based composite materials, which have become the preferred choice for esthetic restorations. The availability of a wide range of color shades has contributed to the improved color matching of resin composite restorations with the natural color of the tooth. Furthermore, the introduction of hybrid and nanofilled resin composites has enhanced the surface finish and texture of restorations, resulting in a more natural appearance [1]. The effectiveness of resin composite restorations is primarily determined by their surface properties and color stability. Nonetheless, the issue of discoloration remains a significant challenge, particularly when the restorations are exposed to the oral environment for an extended period. This discoloration not only compromises the color match of the restoration but also leads to patient discontent, necessitating the extra expense of replacement [2]. The discoloration of restorations can be attributed to either extrinsic or intrinsic factors. Extrinsic factors encompass the buildup of plaque and surface stains, as well as surface or sub-surface changes that lead to superficial degradation or minimal penetration and absorption of staining agents into the outer layer of the resin composite [3]. In contrast, intrinsic discoloration arises from chemical alterations that occur within the material itself. This includes phenomena such as the leaching of unreacted monomers through hydrolysis reactions and the presence of photo-initiator components that were not fully used within the light-curing process [4]. Several factors can influence the extent of color change in resin composites. These include the composition and structure of the resin composite itself, the properties of the filler particles used, the degree of polymerization achieved during curing, and the amount of water absorbed by the material [5]. External factors such as the intake of colored food and drinks, smoking habits, and oral hygiene practices also play significant roles in affecting the color of resin composites [6]. The finishing and polishing steps performed on resin composites can impact the surface quality of the restorations and potentially influence their susceptibility to early discoloration [3]. However, it is worth noting that the influence of polishing on discoloration can vary across various studies, leading to inconsistent findings [3, 7]. Furthermore, surface roughening resulting from wear can have an impact on the gloss of resin composites and, as a result, potentially increase the susceptibility to extrinsic staining [8].

Dentists often receive inquiries from patients regarding the expected lifespan of esthetic restorations and whether their dietary habits can impact the quality and clinical survival of the restorations. It is important to note that the drinking of specific beverages, like coffee and tea, can potentially influence both the esthetic and physical characteristics of resin composites, thus compromising the overall quality of the restoration. Additionally, the high consumption of acidic aerated drinks among young adults and children poses a risk as the acidity of these drinks can be detrimental to the properties of resin composites. The impact of beverages on the characteristics of resin composites could also be directly linked to the frequency and amount of intake [9].

There are several methods available for the removal of surface discoloration from composite restorations. Tooth brushing, polishing techniques, and bleaching protocols are commonly used. Commercially available bleaching agents or ozone, known for its strong oxidizing capacity, have also been utilized in bleaching procedures [3]. While tooth brushing can effectively remove stains, it is a relatively slower process. As a result, faster methods such as polishing or bleaching techniques are often preferred for more efficient stain removal [10]. A thorough review of the available literature indicates a contradiction concerning the impact of beverages on composite restorations and the influence of repolishing on the color change of stained composites [9–12]. The objective of this study was to assess the color stability of six commercially available restorative resin composites (including conventional, bulk-fill, and discontinuous microfiber-reinforced types) after exposure to commonly consumed beverages. This evaluation was conducted using reflection spectrophotometry, employing the CIE (Commission Internationale de I’Eclairage) L* a* b* color system. Additionally, the study aimed to investigate the impact of repolishing on the color change of stained composite materials. The null hypotheses tested in this study were as follows: (1) The type of beverage and resin composite do not have an influence on the staining of composites. (2) The repolishing procedure does not have an influence on the staining of composites.

## Materials and methods

Table 1 provides a list of the resin composites utilized in the study. Twenty five disk-shaped specimens (8 mm diameter & 3 mm thickness) were fabricated from each material using a silicon mold. Sample size calculation was based on previous studies in literature [3, 4, 9]. During the specimen preparation process, a Mylar strip and a 1 mm thick glass slide were placed over the silicone mold. A slight pressure of 5–10 N was applied to the glass slide. This pressure served two purposes: to remove any excess composite material from the mold and to ensure standardization of the specimen thickness and the distance between the specimens and the light curing tip. One operator was preparing all specimens.

**Table 1:** Resin composites used with their composition*

Material (shade)	Manufacturer	Type	Matrix	Filler (wt%)
Filtek™ Universal Restorative (D3)	3 M, St Paul, MN,USA	Nano-filled universal	AUDMA, AFM, DUDMA, and DDDMA	Silica and zirconia 76.5%
SDR flow+ (Universal)	Dentsply, Milford, USA	Bulk fill flowable	TEGDMA, EBPADMA	Barium borosilicate glass 68%
everX Flow (Dentin)	GC Corp, Tokyo, Japan	Short-fibre reinforced flowable	Bis-EMA, TEGDMA, UDMA	70% Short glass fiber & barium glass fillers
G-ænial® A’CHORD (A2)	GC Corp, Tokyo, Japan	Universal composite	Bis-MEPP, UDMA	Barium glass 79%
G-ænial Universal Flo (A2)	GC Corp, Tokyo, Japan	Flowable composite	UDMA, dimethacrylate co-monomers	Barium glass 69%
G-ænial Universal Injectable (A2)	GC Corp, Tokyo, Japan	Flowable composite	Dimethacrylate monomers	Barium glass, silica 69%

* Composition based on manufacturer details. Bis-GMA, bisphenol-A-glycidyl dimethacrylate; TEGDMA, triethylene glycol dimethacrylate; UDMA, urethane dimethacrylate; EBPADMA, Ethoxylated bisphenol A dimethacrylate; AUDMA, Aromatic urethane dimethacrylate; DDDMA, 12-dodecanediol dimethacrylate; Bis-EMA, Ethoxylated bisphenol-A-dimethacrylate; Bis-MPEPP, Bisphenol A polyethoxy methacrylate; AFM, addition-fragmentation monomer; DUDMA, Diurethane dimethacrylate; wt%, weight%

Resin composites were light cured using LED light curing unit (Elipar Deepcure-S, 3 M ESPE, Germany) with an intensity of 1200 mW/cm^2^ for 20 s from both upper and lower surfaces. Before preparing each group, the irradiance of the light curing unit was verified using a calibrator (MARC resin calibrator, BlueLight Analytics, Canada). Following the light curing process, the specimens were polished under running water using 600-grit silicon carbide abrasive paper (CarbiMet, Buehler, Lake Bluff, IL). Once the required thickness was attained, one side of the specimen surface facing the mold, which was the lower surface, was finished and polished with abrasive paper (1000-grit, 2000-grit and 4000-grit FEPA) for 20 s for each at 300 rpm under water cooling using an automatic grinding machine (struers Rotopol-11, Cophenhagen, Denmark) and this side named machine polish. The upper surface of the specimens facing the mylar strip and the glass slide was finished with 1000-grit abrasive paper. Subsequently, these surfaces were polished with a 3 M™ Sof-Lex™ Diamond Polishing System (pre-polishing spiral and diamond-impregnated polishing spiral) for 20 s for each spiral with a slow speed hand piece under water cooling and this side named hand polish.

To ensure uniformity, a digital caliper with an accuracy of 0.01 mm (World Precision Instruments, Sarasota, Florida) was employed to determine the thickness of each specimen. Following the polishing procedure, the specimens were dipped in distilled water and stored at 37 °C for 24 h before the baseline color measurement was taken.

To evaluate the color change in five different beverages, 25 specimens from each material were randomly divided into five groups, according to the used staining solution. Group 1: distilled water (Ph ≈ 7), Group 2: coffee (Nescafe classic, Nestle, Morocco, 3.6 g of coffee powder was diffused in 300 ml of boiling distilled water, Ph ≈ 5), Group 3: red wine (Frontera Cabernet Sauvignon, Chile, Ph ≈ 3.5), Group 4: energy drink (Red Bull GmbH, Ph ≈ 3.3), group 5: coke (Coca-Cola company, Ph ≈ 2.5).

Composite specimens were placed into the containers with both sides being exposed to the staining solution. To avoid the drying up of staining solutions, the specimens were stored in closed containers throughout the duration of the study. The beverages used for staining were renewed on a weekly basis. The specimens were placed inside an incubator set at a temperature of 37 °C, except during solution changes and color measurements.

The color changes (∆E) for all specimens were determined at the baseline, after 84 days of staining and after repolishing. After staining interval, the specimens were washed in distilled water, wiped with a paper towel, and submitted to color assessment from both surfaces. After the color assessment, the stained specimens were repolished and the color measurements were performed again. The repolishing procedure of the machined polished surface was performed with 4000-grit abrasive paper for 20 s and the hand polished surface was performed with the same protocol with 3 M™ Sof-Lex™ Diamond Polishing System.

Color and spectral reflectance measurements were conducted at baseline and after staining using the Konica Minolta VLTCM-700d spectrophotometer (Tokyo, Japan). It was not possible to blind the operator during these measurements. The measurements were performed on the CIE Lab color scale. The spectrophotometer had an aperture size of Ø 3 mm, and the illuminating and viewing configuration followed the CIE diffuse/80 geometry with the inclusion of the specular component (SCI) geometry.

To calculate the color difference (∆E), the mean values of ∆L, ∆a, and ∆b for each specimen were utilized in the following formula:

ΔE= [(ΔL)^2^+(Δa)_2_+(Δb)^2^]^1/2^.

where, ∆L, ∆a, ∆b are differences in L a b values between baseline and after staining.

L* represents the lightness of the color (range between black and white), a* gives information about the color position on the green and red axis, b* represents the color position on yellow and blue axis. Based on previous studies [13, 14], a perceptible color change that ΔE > 1 will be referred to as acceptable up to the value ΔE = 3.3.

A statistical software (IBM SPSS Statistics v28.0, IBM, USA) was used for all the statistical analyses. Levene’s test of equality was used to test the distribution of variance. To assess the disparities in color variation values among the tested specimens across different beverages and polishing technique, 3-way analysis of variance (ANOVA) was used at a significance level of p < 0.05. Followed by a 2-way ANOVA and Tukey’s multiple comparisons as a post hoc analysis to compare each material’s response to staining solutions and repolishing procedures.

## Results

**Fig. 1:**
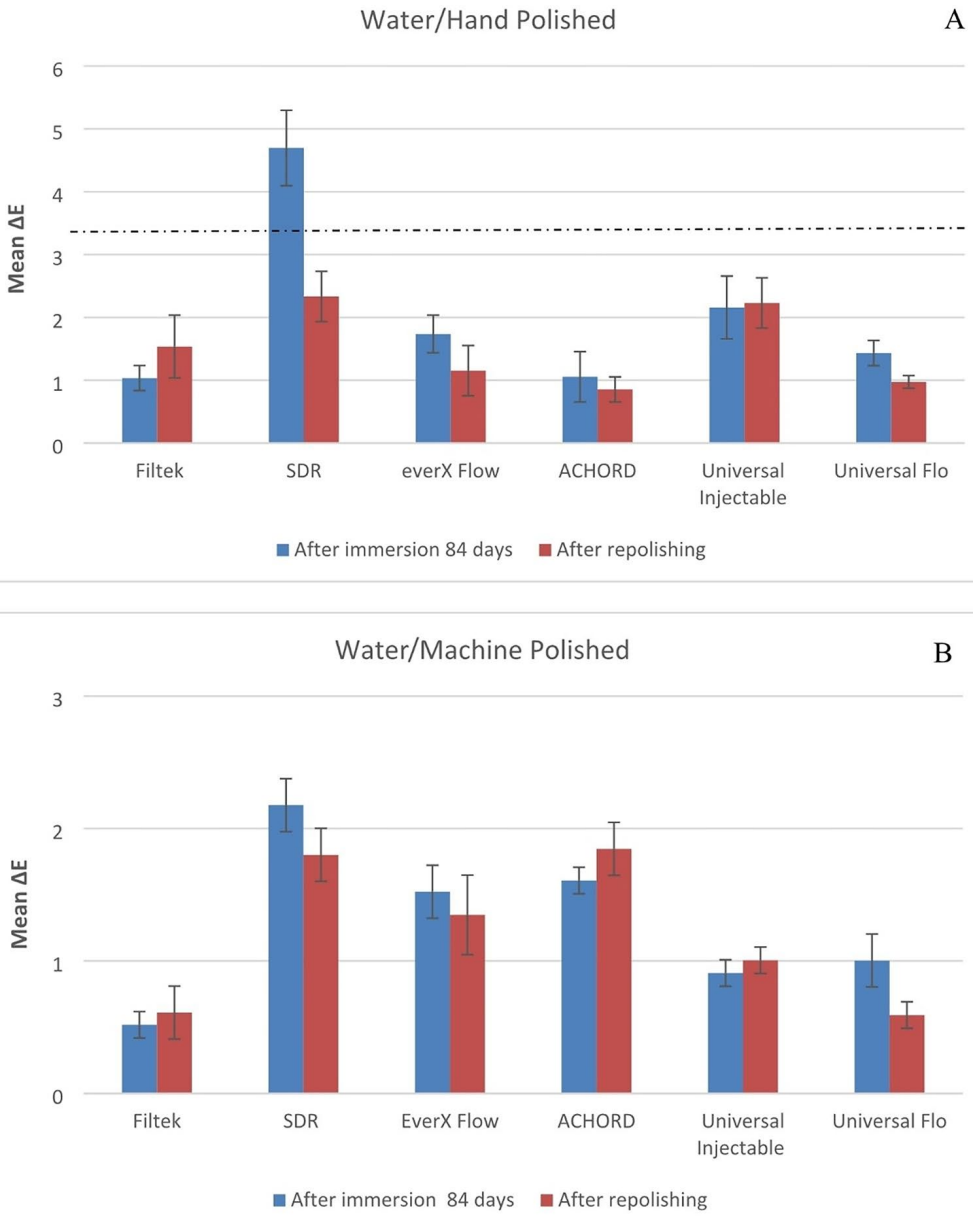
Mean color differences (∆E) of the tested resin composites after 84 days of immersion in water and after repolishing. (**A**) Hand polished surface. (**B**) Machine polished surface. Values exceeding the dotted line indicate clinical unacceptability (∆E ≥ 3.3)

**Fig. 2:**
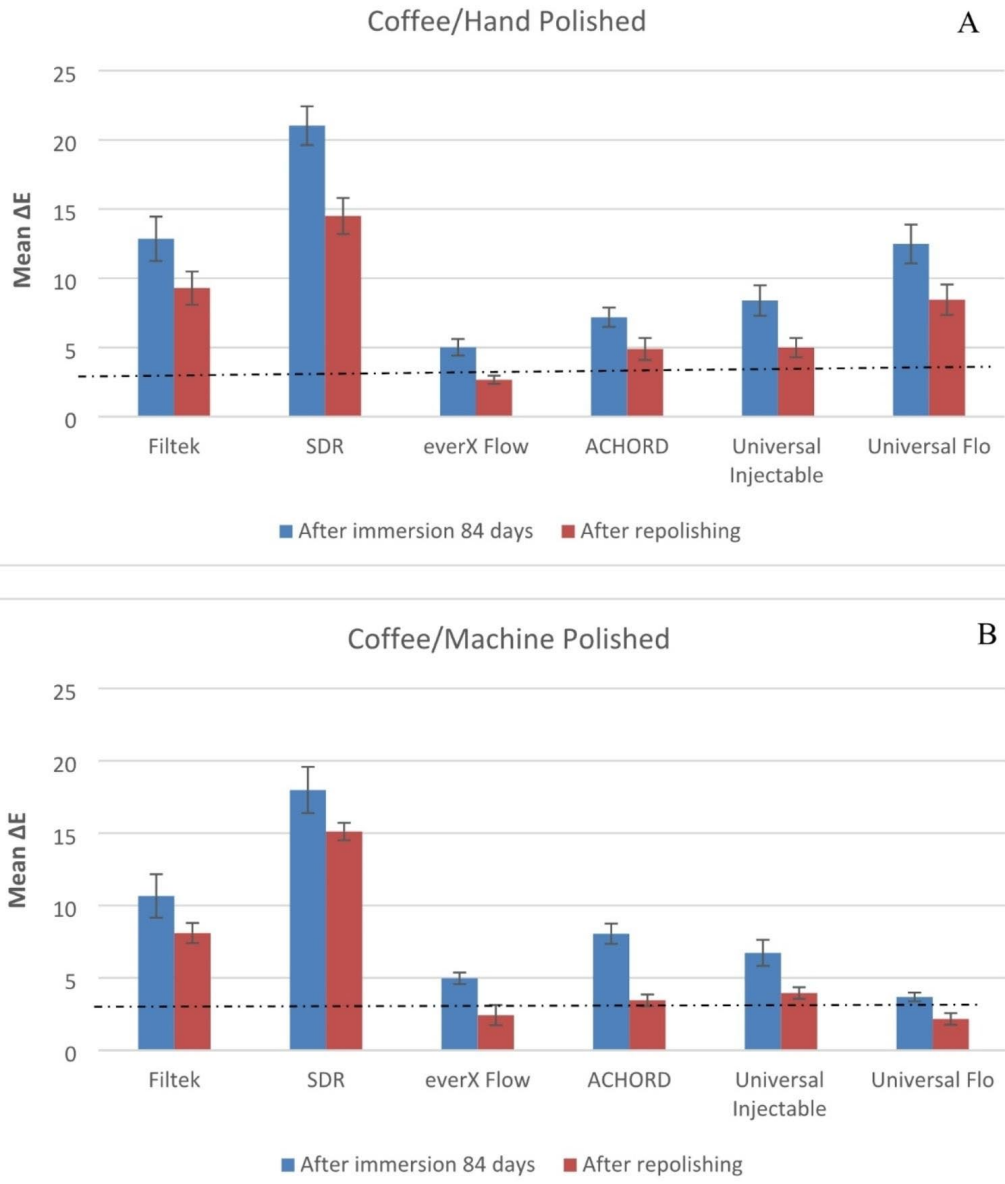
Mean color differences (∆E) of the tested resin composites after 84 days of immersion in Coffee and after repolishing. (**A**) Hand polished surface. (**B**) Machine polished surface. Values exceeding the dotted line indicate clinical unacceptability (∆E ≥ 3.3)

**Fig. 3:**
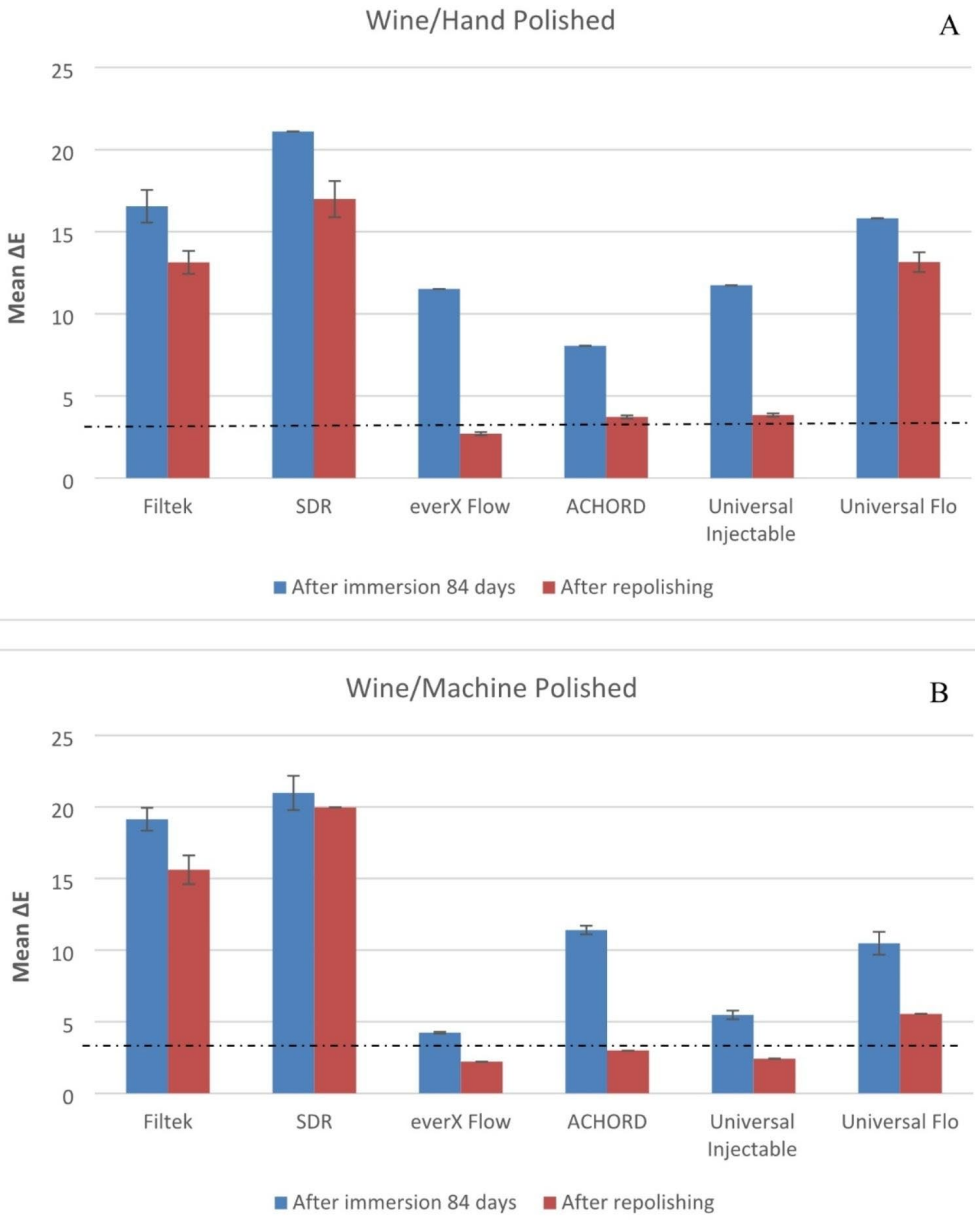
Mean color differences (∆E) of the tested resin composites after 84 days of immersion in Red wine and after repolishing. (**A**) Hand polished surface. (**B**) Machine polished surface. Values exceeding the dotted line indicate clinical unacceptability (∆E ≥ 3.3)

**Fig. 4:**
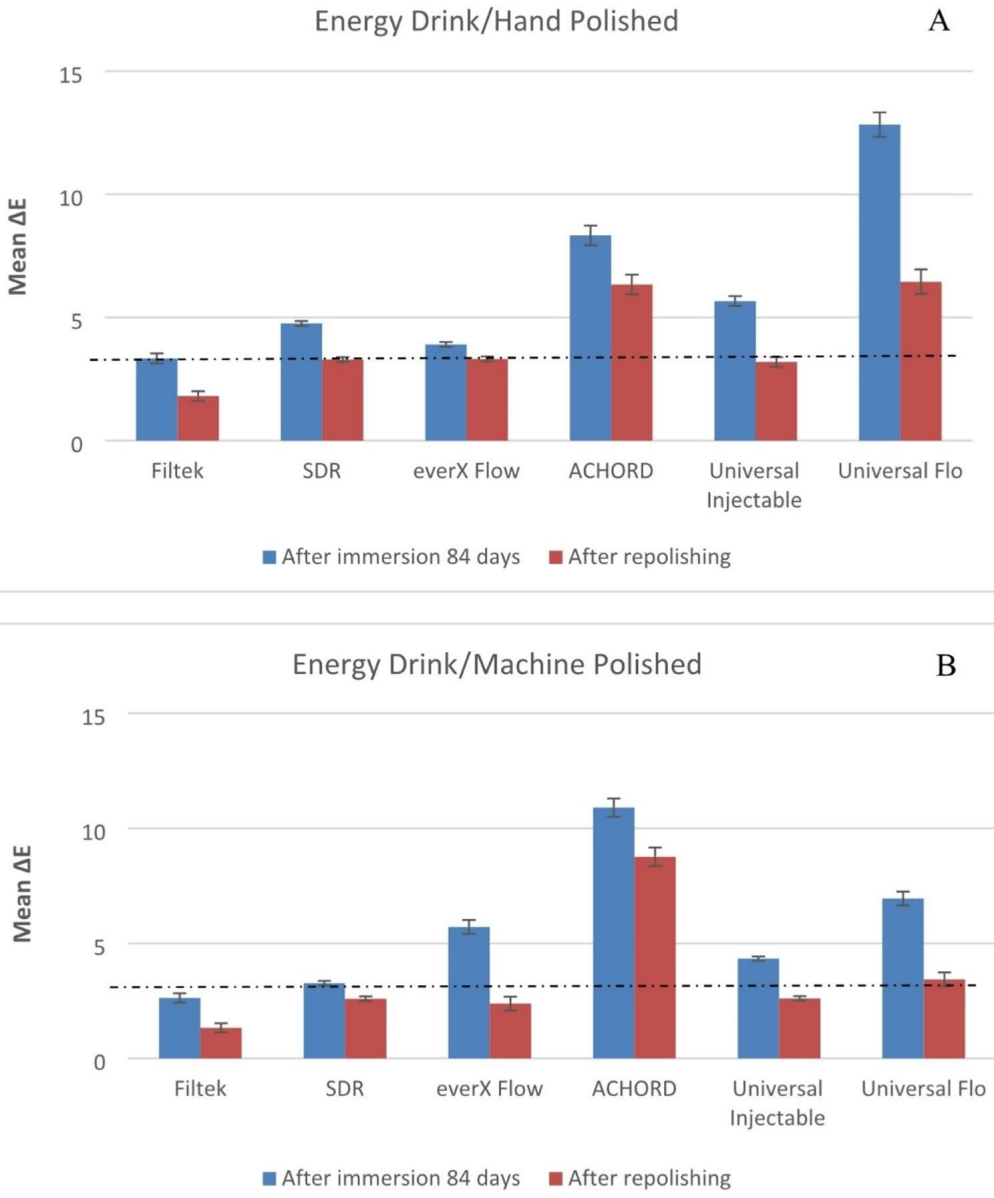
Mean color differences (∆E) of the tested resin composites after 84 days of immersion in Energy drink and after repolishing. (**A**) Hand polished surface. (**B**) Machine polished surface. Values exceeding the dotted line indicate clinical unacceptability (∆E ≥ 3.3)

**Fig. 5:**
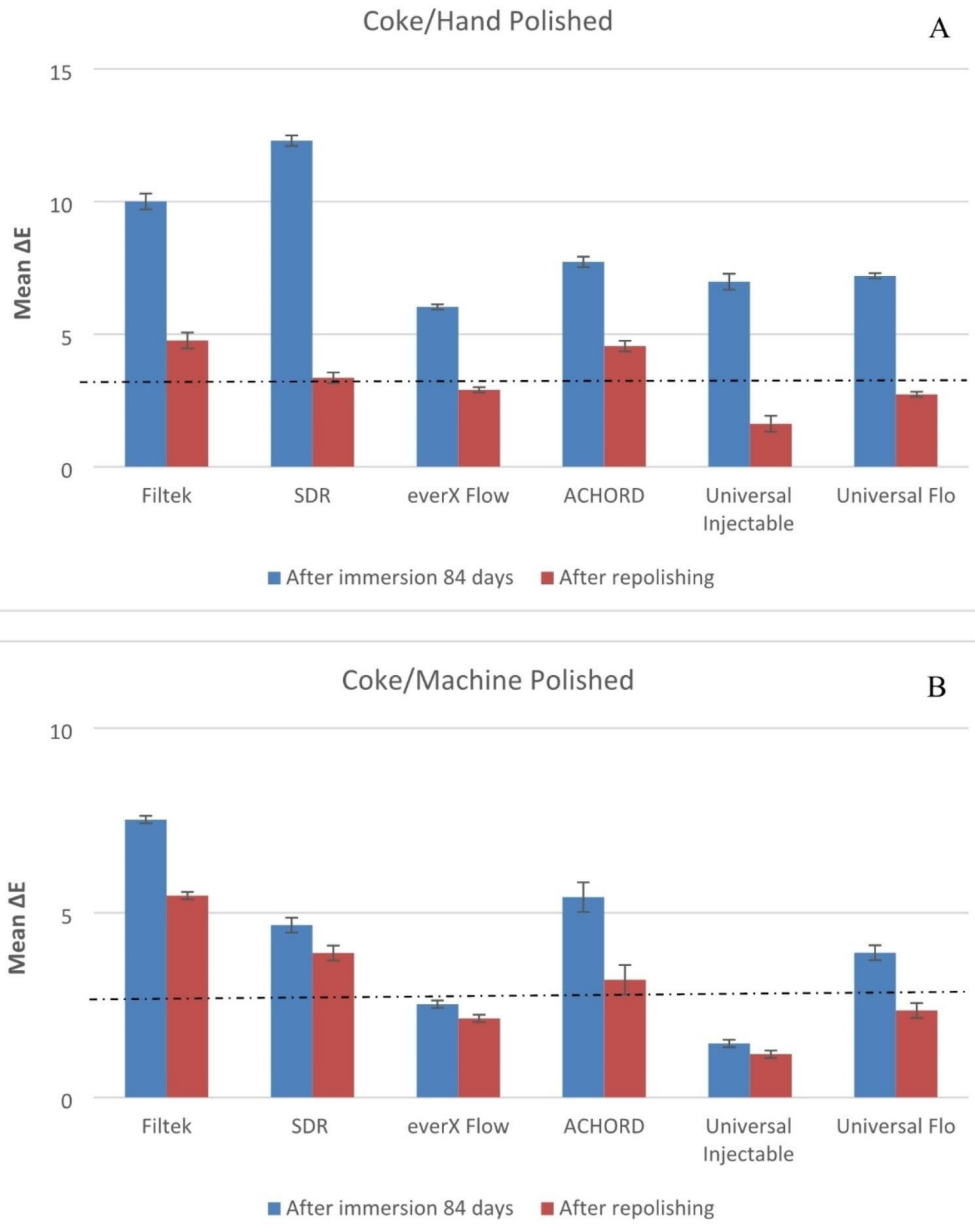
Mean color differences (∆E) of the tested resin composites after 84 days of immersion in coke and after repolishing. (**A**) Hand polished surface. (**B**) Machine polished surface. Values exceeding the dotted line indicate clinical unacceptability (∆E ≥ 3.3)

Both material type and staining solution have a significant effect on color stability of specimens (p < 0.05). The means of the change in color of the machine polish or hand polish specimens after 84 days in storage and after repolishing are presented in Figs. 1, 2, 3, 4 and 5.

The mean ∆E values of the specimens immersed in coffee and wine were found to be significantly higher (p < 0.05) compared to the other beverages. SDR flow + in coffee and wine presented the highest ∆E when compared to other tested materials (p < 0.05). While G-ænial Universal Flo and A’CHORD exhibited the highest ∆E values after being immersed in an energy drink (p < 0.05).

The influence of polishing technique (machine & hand) on staining was found to be dependent on the material used. Machine polishing of SDR flow+, everX Flow, G-ænial Universal Flo, and G-ænial Universal Injectable composites led to significantly lower ∆E values (p < 0.05) compared to hand polishing technique. On the other hand, no differences were observed for other composites.

After staining of the composites, repolishing (machine & hand) was able to reduce the ∆E value (p < 0.05).

## Discussion

The discoloration of composite, particularly at the margins, is a significant factor contributing to the esthetic failure of restorations. This can often lead to the replacement of restorations in esthetic areas [15]. The discoloration process not only concerns patients but also dentists, as it requires additional time and resources to address. Consequently, it poses concerns for both dentist and patient and can result in unnecessary expenses. In our study, the color stability of the specimens was significantly affected by both the type of resin composite and the staining solution used. Thus, the first null hypotheses were accepted.

The susceptibility of resin composites to staining can be influenced by various factors, such as the degree of monomer conversion (DC%), the degree of water sorption, and the hydrophilicity of the resin matrix [16]. When a resin composite has the ability to absorb water, it is also prone to absorbing other fluids, which can contribute to discoloration [3]. The varying compositions of resin composites lead to differences in their susceptibility to staining, primarily associated with their water uptake properties. It is believed that water serves as a medium for the penetration of stains into the resin matrix [17]. The filler particles within resin composites do not absorb water into the bulk of the material but can absorb water on their surface. As a result, a higher proportion of resin matrix in the composites leads to increased water absorption and weaker bonding between the resin matrix and filler particles. This can result in the formation of microcracks within the resin matrix due to swelling and plasticizing effects, as well as the creation of interfacial gaps between the filler and resin matrix. These factors facilitate the penetration of stains and subsequent discoloration of the restorations [17].

The assessment of discoloration can be performed using visual or instrumental techniques. In our study, we employed spectrophotometry, which eliminates subjective interpretation in visual color comparisons. Spectrophotometry has been recognized as a reliable technique in dental materials studies [3, 18]. The color change (∆E) value provides a measure of the relative color changes that an observer may perceive in the materials after immersion or over time. Therefore, ∆E is a more meaningful metric than the individual ∆L, ∆a, and ∆b values [19].

In our study, we assessed the color stability of resin composites by immersing them in five commonly consumed beverages (water, coffee, red wine, energy drink, and cola) for a duration of 84 days. It is worth noting that a comprehensive review of literature conducted over the past decade indicated that the maximum immersion time used in previous studies was typically 30 days [20–22]. To simulate realistic beverage consumption patterns, we considered the average time required to consume a cup of coffee, which is approximately 15 min. Additionally, considering the average coffee consumption of three cups per day among coffee drinkers, our 12-week immersion period effectively simulated the equivalent of consuming the beverage over a period of seven years [23].

**Fig. 6:**
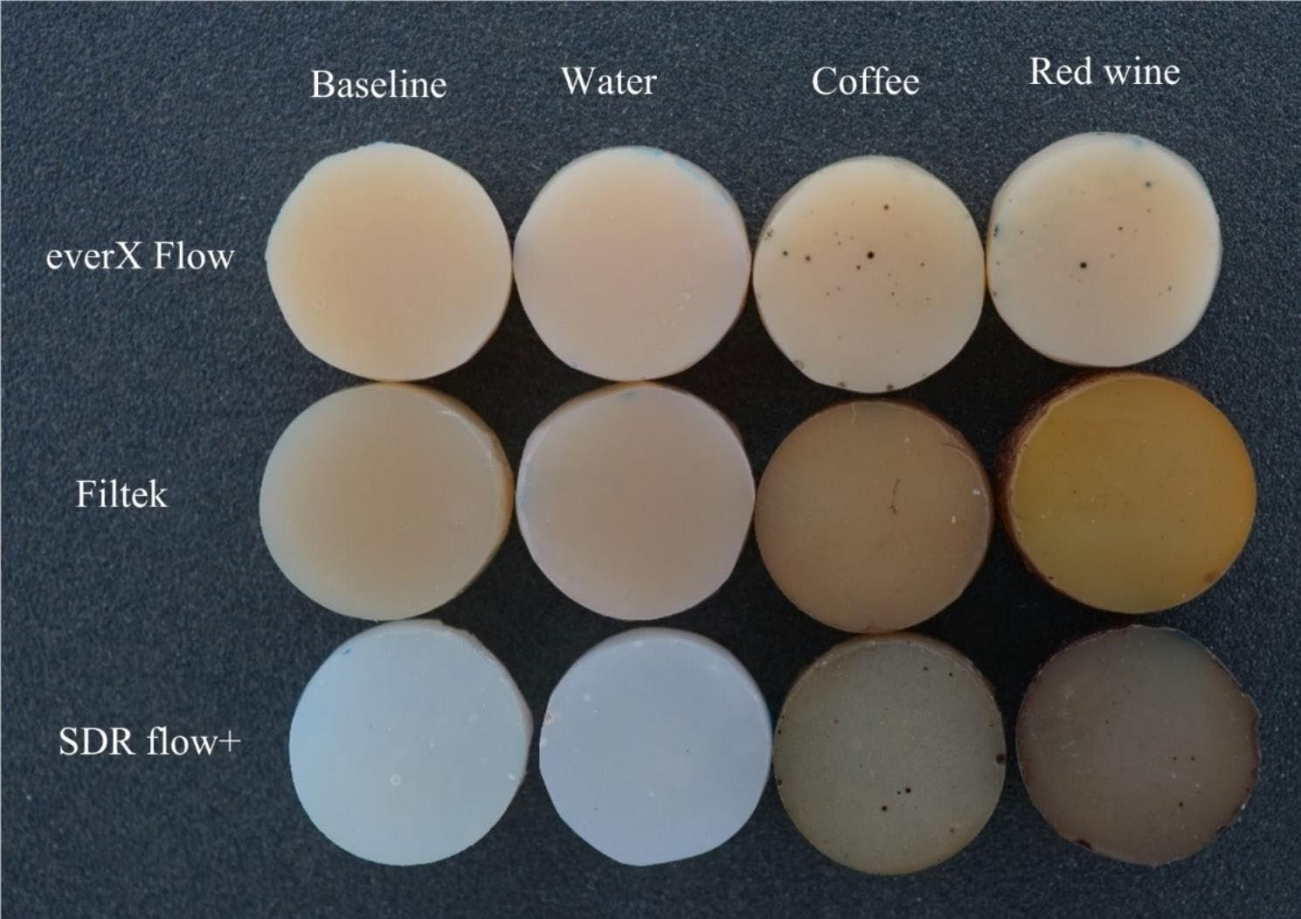
Photographs of representative stained specimens from discontinuous fiber-reinforced (everX Flow), conventional (Filtek) and bulk-fill (SDR flow+) resin composites

Consistent with previous research findings [3, 21, 24], our study confirmed that coffee and red wine led to the most pronounced discoloration of composites, which was readily observable with the naked eye (Fig. 6). The staining effects of coffee and wine can be attributed to the absorption of their colorants into the organic phase of the composites [24]. Surprisingly, coke and energy drink which contain acids (phosphoric & citric) with a pH range of 2.5–3.5, did not show a strong association with color changes in the composites compared to coffee and wine. The behavior of acids can vary in terms of promoting dissolution, water uptake, and material erosion. Bagheri et al., and Ertas et al., have suggested that the absence of yellow colorants in coke results in less discoloration compared to the staining caused by coffee [17, 25].

Numerous studies have indicated that ∆E values in the range of 1 to 3 are noticeable to the naked eye, while ∆E values exceeding 3.3 are considered clinically unacceptable [26, 27]. Taking these concepts into consideration, the resin composites examined in the current study exhibited undesirable color change after being stored in staining solutions for a duration of 84 days, with the exception of water. Among the tested materials, only SDR flow + in water demonstrated color change values exceeding 3.3, indicating the need for surface coverage in the patient’s mouth. Due to the presence of TEGDMA in its composition (Table 1), SDR flow + may have a higher tendency to release monomers into aqueous environments compared to resin composites based on Bis-GMA, Bis-EMA and UDMA. Consequently, SDR flow + may exhibit more noticeable color changes [28, 29]. In contrast, everX Flow also contains TEGDMA in its composition. Nevertheless, it showed a reduced susceptibility to staining when compared to SDR flow+ (Figs. 1, 2, 3, 4, 5 and 6). It is challenging to determine the exact significance of specific dental formulations over others when it comes to color stability. The affinity of the resin matrix for staining is also influenced by its DC% [16]. The level of unreacted monomers present is directly related to the DC%. A higher degree of monomer conversion indicates a lower amount of unreacted monomers, reduced water uptake, and enhanced color stability. Based on the available literature [30], it has been reported that SDR flow + has a lower DC% (59) compared to everX Flow (63). This difference in DC% may account for the variations in color stability observed between the two materials. Although, the microfiber-reinforced composite is intended to be eventually covered by another more esthetic composite according to manufacturer instructions, still it has comparably good color stability when viewed against other tested bulk fill and conventional resin composites. The variations in color stability observed among the tested materials could also be attributed to the differences in filler/resin ratio (Table 1). Previous studies have reported that resin composites with a low filler/resin ratio, which often corresponds to a lower viscosity, may exhibit reduced polishability and color stability [31–33]. Moreover, the observed color change can also be attributed to the silane agent used. Silanization of inorganic particles is known to contribute to discoloration due to the high water sorption capacity of silane [34, 35]. In addition to the previously mentioned explanation, the shade of the resin composite is another aspect influencing composite staining. Darker shades generally exhibit improved color matching capabilities due to the presence of pigments. As a result, universal shades, which lack these pigments, may experience a more significant degree of color change [36]. Consistent with the findings, both SDR flow + and Filtek, which have a universal shade, exhibited significant color changes compared to other resin composites.

Polishing techniques have been mentioned in the literature as another factor that may influence color stability. However, there is no consensus regarding the impact of surface treatment on color change caused by staining. Some studies suggest that smoother composite surfaces do not necessarily exhibit greater resistance to staining, while others indicate that the absence of proper polishing increases the susceptibility of specimens to staining [3, 7, 35]. The findings on the relationship between surface treatment and color stability are still inconclusive and further research is needed to clarify this matter. The absence of proper polishing may result in the retention of a resin-rich surface layer on the composite material. This can contribute to higher color changes when the material is exposed to staining solutions. In this study, one surface of all the composite specimens was polished using the Sof-Lex™ Polishing System to closely mimic clinical conditions. The other surface of the specimens, which faced the mylar strip and glass slide, underwent a highly polished laboratory technique using 4000-grit polishing. However, contrary to expectations, the results showed that the polishing technique did not have a significant effect on the color change of the composite specimens for all materials. This finding aligns with the study conducted by Imamura et al., who also demonstrated that the polishing technique did not strongly influence composite discoloration [36]. After staining occurs, repolishing and bleaching procedures are considered as potential whitening techniques that can partially or completely remove stains [10]. According to Fontes et al., the pigmented layer of the composite (approximately 40 μm) or the absorbed stains could theoretically be eliminated through polishing [37]. Türkün LS & Türkün M also suggested that in-office bleaching and repolishing procedures can partially remove discoloration of composites [38]. In the present study, repolishing was found to effectively reduce the color change values in all composite specimens (Figs. 1, 2, 3, 4 and 5). Thus, the second null hypothesis was also accepted. However, some ∆E values were still higher than 3.3, which are not acceptable clinically. Our findings are consistent with prior studies that have demonstrated the effectiveness of repolishing composites after staining with various solutions in reducing the ∆E values to some degree [3, 38, 39].

In vitro studies inherently possess methodological limitations when evaluating color stability. In this particular study, our objective was to simulate the long-term impact of oral environmental factors within a short timeframe of 84 days, aiming to predict the clinical performance of the resin composites. It is important to note that within the oral cavity, the impact of factors such as heat from hot drinks and food, saliva, and other fluids may have a more pronounced effect. Moreover, the mastication process can alter the surface roughness of the composite, allowing discoloration factors and deposits to remain on rough surfaces for extended periods. Additionally, the intermittent contact between dental structures and restorative materials with staining agents, coupled with mechanical wear, can exacerbate the discoloration process.

## Conclusions

The color stability of the tested resin composites was influenced by all the beverages used, and the variations in color stability changed depending on the specific material utilized. Dentists should possess awareness regarding the chemical interactions that occur between different beverages and various types of resin composites. Additionally, repolishing serves as an effective technique for eliminating surface discoloration in composite restorations.

## Data Availability

The datasets used and/or analysed during the current study available from the corresponding author on reasonable request.

## References

[1] de Oliveira AG, Rocha RS, Spinola MDS, Batista GR, Bresciani E, Caneppele TMF (2023). Surface smoothness of resin composites after polishing-A systematic review and network meta-analysis of in vitro studies. Eur J Oral Sci.

[2] Al-Asmar AA, Ha Sabrah A, Abd-Raheam IM, Ismail NH, Oweis YG (2023). Clinical evaluation of reasons for replacement of amalgam vs composite posterior restorations. Saudi Dent J.

[3] Garoushi S, Lassila L, Hatem M, Shembesh M, Baady L, Salim Z, Vallittu P (2013). Influence of staining solutions and whitening procedures on discoloration of hybrid composite resins. Acta Odontol Scand.

[4] Barutcigil Ç, Yıldız M (2012). Intrinsic and extrinsic discoloration of dimethacrylate and silorane based composites. J Dent.

[5] Paolone G, Formiga S, De Palma F, Abbruzzese L, Chirico L, Scolavino S, Goracci C, Cantatore G, Vichi A (2022). Color stability of resin-based composites: staining procedures with liquids-A narrative review. J Esthet Restor Dent.

[6] Lepri CP, Palma-Dibb RG (2012). Surface roughness and color change of a composite: influence of beverages and brushing. Dent Mater J.

[7] Çakmak G, Oosterveen-Rüegsegger AL, Akay C, Schimmel M, Yilmaz B, Donmez MB. Influence of polishing technique and coffee thermal cycling on the surface roughness and color stability of additively and subtractively manufactured resins used for definitive restorations. J Prosthodont. 2023 Jul;8. 10.1111/jopr.13730. Epub ahead of print. 10.1111/jopr.13730 37421940

[8] de Melo TP, Delgado A, Martins R, Lassila L, Garoushi S, Caldeira J, Azul AM, Vallittu P (2022). Can Specular gloss measurements predict the effectiveness of Finishing/Polishing protocols in Dental polymers? A systematic review and Linear mixed-effects prediction model. Oper Dent.

[9] Choi JW, Lee MJ, Oh SH, Kim KM (2019). Changes in the physical properties and color stability of aesthetic restorative materials caused by various beverages. Dent Mater J.

[10] Abd Elhamid M, Mosallam R (2010). Effect of bleaching versus repolishing on colour and surface topography of stained resin composite. Aust Dent J.

[11] Ugurlu M (2022). Effect of repolishing on the discoloration of indirect composite block, nanohybrid, and microhybrid resin composites. Eur Oral Res.

[12] Paolone G, Mazzitelli C, Boggio F, Breschi L, Vichi A, Gherlone E, Cantatore G (2023). Effect of different Artificial Staining procedures on the Color Stability and Translucency of a Nano-Hybrid Resin-based composite. Mater (Basel).

[13] Villalta P, Lu H, Okte Z, Garcia-Godoy F, Powers JM (2006). Effects of staining and bleaching on color change of dental composite resins. J Prosthet Dent.

[14] Fay RM, Servos T, Powers JM. Color of restorative materials after staining and bleaching. Oper Dent. 1999 Sep-Oct;24(5):292-6. 10823076

[15] Endo Hoshino IA, Fraga Briso AL, Bueno Esteves LM, Dos Santos PH, Meira Borghi Frascino S, Fagundes TC (2022). Randomized prospective clinical trial of class II restorations using flowable bulk-fill resin composites: 4-year follow-up. Clin Oral Investig.

[16] Hyun HK, Ferracane JL (2016). Influence of biofilm formation on the optical properties of novel bioactive glass-containing composites. Dent Mater.

[17] Bagheri R, Burrow MF, Tyas M (2005). Influence of food-simulating solutions and surface finish on susceptibility to staining of aesthetic restorative materials. J Dent.

[18] He J, Garoushi S, Säilynoja E, Vallittu P, Lassila L (2021). Surface Integrity of Dimethacrylate Composite resins with low shrinkage comonomers. Mater (Basel).

[19] Yannikakis SA, Zissis AJ, Polyzois GL, Caroni C (1998). Colour stability of provisional resin restorative materials. J Prosthet Dent.

[20] Korać S, Ajanović M, Džanković A, Konjhodžić A, Hasić-Branković L, Gavranović-Glamoč A, Tahmiščija I (2022). Color Stability of Dental Composites after immersion in beverages and performed whitening procedures. Acta Stomatol Croat.

[21] Poggio C, Ceci M, Beltrami R, Mirando M, Wassim J, Colombo M (2016). Color stability of esthetic restorative materials: a spectrophotometric analysis. Acta Biomater Odontol Scand.

[22] Nasim I, Neelakantan P, Sujeer R, Subbarao C (2010). Colour stability of microfilled, microhybrid and nanocomposite resins-An in vitro study. J Dent.

[23] Guler AU, Yilmaz F, Kulunk T, Guler E, Kurt S (2005). Effects of different drinks on stainability of resin composite provisional restorative materials. J Prosthet Dent.

[24] Lopes-Rocha L, Mendes JM, Garcez J, Sá AG, Pinho T, Souza JCM, Torres O (2021). The Effect of different dietary and therapeutic solutions on the Color Stability of Resin-Matrix composites used in Dentistry: an in Vitro Study. Mater (Basel).

[25] Ertaş E, Güler AU, Yücel AC, Köprülü H, Güler E (2006). Color stability of resin composites after immersion in different drinks. Mater J.

[26] Noie F, O’keefe KL, Powers JM (1995). Color stability of resin cements after accelerated aging. Int J Prosthodont.

[27] Ruyter IE, Nilner K, Moller B (1987). Color stability of dental composite resin materials for crown and bridge veneer. Dent Mater.

[28] Silva MF, Dias MF, Lins-Filho PC, Silva CH, Guimarães RP (2020). Color stability of Bulk-Fill composite restorations. J Clin Exp Dent.

[29] Paolone G, Mandurino M, Scotti N, Cantatore G, Blatz MB (2023). Color stability of bulk-fill compared to conventional resin-based composites: a scoping review. J Esthet Restor Dent.

[30] Lassila L, Säilynoja E, Prinssi R, Vallittu P, Garoushi S (2019). Characterization of a new fiber-reinforced flowable composite. Odontology.

[31] Uçtaşli MB, Arisu HD, Omürlü H, Eligüzeloğlu E, Ozcan S, Ergun G (2007). The effect of different finishing and polishing systems on the surface roughness of different composite restorative materials. J Contemp Dent Pract.

[32] Sulaiman TA, Rodgers B, Suliman AA, Johnston WM (2021). Color and translucency stability of contemporary resin-based restorative materials. J Esthet Restor Dent.

[33] Lassila L, Säilynoja E, Prinssi R, Vallittu PK, Garoushi S (2020). The effect of polishing protocol on surface gloss of different restorative resin composites. Biomater Investig Dent.

[34] Mailart MC, Rocha RS, Contreras SCM, Torres CRG, Borges AB, Caneppele TMF (2018). Effects of artificial staining on bulk-filled resin composites. Am J Dent.

[35] Imamura S, Takahashi H, Hayakawa I, Loyaga-Rendon PG, Minakuchi S (2008). Effect of filler type and polishing on the discoloration of composite resin artificial teeth. Dent Mater J.

[36] Arregui M, Giner L, Ferrari M, Vallés M, Mercadé M (2016). Six-month color change and water sorption of 9 new-generation flowable composites in 6 staining solutions. Braz Oral Res.

[37] Fontes ST, Fernández MR, de Moura CM, Meireles SS (2009). Color stability of a nanofill composite: effect of different immersion media. J Appl Oral Sci.

[38] Türkün LS, Türkün M (2004). Effect of bleaching and repolishing procedures on coffee and tea stain removal from three anterior composite veneering materials. J Esthet Restor Dent.

[39] Mundim FM, Garcia Lda F, Pires-de-Souza Fde C (2010). Effect of staining solutions and repolishing on color stability of direct composites. J Appl Oral Sci.

